# The Effect of Silane Coupling Agent on the Texture and Properties of In Situ Synthesized PI/SiO_2_ Nanocomposite Film

**DOI:** 10.3390/nano12020286

**Published:** 2022-01-17

**Authors:** Jindong Huang, Hong Chen, Guanglu Zhang, Xiaowei Fan, Juncheng Liu

**Affiliations:** 1 School of Materials Science and Engineering, Tiangong University, Tianjin 300387, China; jdhuang@tiangong.edu.cn (J.H.); chen393994@gmail.com (H.C.); zgltg@tiangong.edu.cn (G.Z.); xiaowei_fan@126.com (X.F.); 2 School of Physical Science and Technology, Tiangong University, Tianjin 300387, China; 3 Tianjin SYP Engineering Glass Co., Ltd., Tianjin 300402, China

**Keywords:** silane coupling agent, polyimide, composite film, transmittance, mechanical properties, radiation resistance

## Abstract

PI/SiO_2_ composite films have been prepared by using in situ polymerization. The influences of the dosage of silane coupling agent (KH-560) on the structure and performance of PI/SiO_2_ composite film have been investigated. The results show that in the components without KH-560, the addition of SiO_2_ decreases the transmittance of the sample. Compared to the same SiO_2_ doping amount, the transmittance in the visible light range of the sample using KH-560 is higher than that of the sample without KH-560. After adding KH-560, the tensile strength, the elastic modulus the elongation at break of the sample have largely changed. The thermal stability and the ability to resist ultraviolet radiation of the composite film first increases and then decreases. Furthermore, the optimal dosage of KH-560 is 3%. Moreover, the addition of KH-560 has little effect on the transmittance of the PI/SiO_2_ composite films before and after UV irradiation.

## 1. Introduction

Organic–inorganic nanocomposite materials not only have the advantages of organic polymer materials, but also have the characteristics of inorganic materials, which makes the organic–inorganic nanocomposite materials become an important research topic in the fields of microelectronics, electric power and aerospace in recent years [1,2,3]. Among various organic/inorganic nanocomposite materials, polyimide/silica (PI/SiO_2_) nanocomposite film has become a research hot pot due to its excellent optical permeability, mechanical properties and radiation resistance [4,5,6,7,8].

As far as PI/SiO_2_ nanocomposites are concerned, the organic component polyimide and the inorganic component silica are thermodynamically incompatible. This incompatibility is not beneficial to the dispersion of inorganic particles in PI. Moreover, the abundantly active hydroxyl groups on the surface of SiO_2_, highly hydrophilic, are easy to form agglomerates or secondary aggregation, which is not conducive to its dispersion in the material [9,10]. These factors are undoubtedly very unfavorable for the synthesis of PI/SiO_2_. Currently, the sol-gel method has been regarded as the most common method for preparing PI/SiO_2_ nanocomposites. However, during the sol-gel reaction process, a certain amount of water and catalyst need to be added, which may cause the residue of some impurities and affect the performance of the film [11,12,13,14]. For improving the process and obtain materials with better comprehensive performance, the in situ polymerization method came into being. The advantage of the in situ polymerization approach is that the size and shape of SiO_2_ can be controlled. The disadvantage is that the free energy of the nanoparticle interface is relatively large, the interaction between the particles is strong, and agglomeration is prone to occur during the polymerization reaction [15,16,17,18,19,20]. In recent years, silane coupling agents have played an increasingly major role in the development and application of organic–inorganic nanocomposites. Silane coupling agents have a special molecular structure. One end of the functional group reacts with inorganic substances, and the other end of the functional group reacts with organic substances. The surface of nanoparticles can be treated by the reasonable use of silane coupling agent before monomer polymerization. So the surface free energy of nanoparticles will decrease and the ability of agglomeration will be reduced. Furthermore, nanoparticles can be uniformly dispersed in PI, increasing the contact with PI. Thus, a bridge of two phases is established, and the combination of the two phases in the nanometer range is realized. However, the influence of silane coupling agent on the structure and performances of PI/SiO_2_ films are rarely reported.

Herein, PI/SiO_2_ composite film has been prepared via in situ polymerization by using KH-560 as the silane coupling agent to decorate the surface of SiO_2_ (with a particle size of about 15 nm), and using 2,2’-bistrifluoromethylbenzidine (TFDB) and 4,4’(hexafluoroisopropyl) diphthalic anhydride (6FDA) as the monomer raw material. The influences of the coupling agent on the structure, mechanical, optical performances and radiation resistance of the composite film have been studied. The results show that a certain addition of KH-560 is beneficial to improve the transmittance, thermal stability and mechanical properties of PI/SiO_2_ films. Furthermore, the optimal addition of KH-560 is 3%.

## 2. Materials and Methods

### 2.1. Synthesis

6FDA, SiO_2_ (~15 nm), and TFDB were bought from Shanghai Macklin Biochemical Technology Co., Ltd. (Shanghai, China). KH-560 (the molecular structure is shown in Figure S1) and N, N’-dimethylacetamide (DMAc) were from Shanghai Jingchun Biochemical Technology Co., Ltd (Shanghai, China).

The SiO_2_ nanoparticles, the diamine and a certain quality of dianhydride were weighed and put into a beaker, further dried in an oven (120 °C for 2 h, 120 °C for 4 h and 180 °C for 4 h), respectively. Furthermore, the molar ratio of diamine to dianhydride is about 1: 1.02. The sample, raw material ratio and the schematic diagram were displayed in Table S1 and Figure S2.

A certain amount of DMAc solvent was added into the dried SiO_2_ beaker. The above mixed solution was stirred until a homogeneous solution was obtained at room temperature. Solutions with 1%, 3%, 6% and 10% KH-560 was added into the above mixed solution and treated by ultrasonic for 2 h. Then, the dried diamine was added into the above solution and stirred until a transparent solution was formed. Subsequently, the dried dianhydride was added into the beaker under a nitrogen atmosphere and kept at 0 °C. When the added amount of dianhydride reached the equivalent point, the whole viscosity of the mixed solution abruptly improved, and the phenomenon of Rod Climbling occurred [18]. In order to facilitate laying the film, DMAc can be properly added to control the solid content at ~10%. Furthermore, the solution was stirred and treated by ultrasonic treatment at 25 °C for 24 h until a transparent solution was obtained, which was maintained at 0 °C for 4 h.

The dried 5 cm × 5 cm glass plate was prepared. Then, a certain amount of polyamide acid/SiO_2_ (PAA/SiO_2_) gel was homogeneously coated on the glass plate with a glass rod. Furthermore, the thickness of the gel was controlled at 20–25 μm. The sample was put in an oven (40 °C, 2 h). The film would be removed after the solvent evaporates sufficiently. A thermal imidization was treated via a stepped heating method, specifically, 60 °C for 1 h, 70 °C for 1 h, 80 °C for 1 h, 100 °C for 1 h, 200 °C for 1 h, 300 °C for 1 h and 350 °C for 1 h. The thermally imidized samples were taken out and placed at room temperature, which was dipped in a water bath for ~10 min. After being isolated from the glass plate, the film was dipped in an acetone solution for ~5 min and dried in an oven (80 °C, 10 min). After that, the PI/SiO_2_ composite films were obtained. In addition, the PI/SiO_2_ films with 1%, 3%, 6% and 10% addition of KH-560 were named as PIS10-560-1, PIS10-560-3, PIS10-560-6 and PIS10-560-10, respectively.

### 2.2. Characterization

Fourier transform infrared spectrometer (FTIR, Nicolet iS50, Thermo Fisher, Waltham, MA, USA) was used to measure the compound structure and functional group composition of the sample. The scanning speed was 800 nm min^−1^ and the test range was 400~4000 nm^−1^. A scanning electron microscope (SEM, GeminiSEM 500, ZEISS, Oberkohen, Germany) was used to characterize the microscopic morphology of the sample. A comprehensive thermal analyzer (TGA, STA 449F5, NETZSCH, Bavaria, Germany) was used to analyze the thermal weight loss of the sample. The measurement environment was nitrogen atomsphere, and the temperature rise rate is 10 °C min^−1^. The transmittance of the sample was measured with an ultraviolet-visible-near-infrared photometer (UH4150, HITACHI, Tokyo, Japan). The measurement wavelength range was 300 nm–800 nm. The material universal testing machine (AGS-X 50N, SHIMADZU, Kyoto, Japan) was used to measure the main mechanical properties of the sample, including elongation at break, tensile strength and elastic modulus. The sample can be cut into a dumbbell shape of 5 cm × 1 cm. The drawing speed is 10 mm min^−1^. Ultraviolet lamp (HJ-1402, Cnlight, Guangdong Province, Foshan, China) was used to simulate the space irradiation environment. The PIS10, PIS10-560-1, PIS10-560-3, PIS10-560-6 and PIS10-560-10 composite films were continuously irradiated for 288 h. The ambient temperature, the humidity, the irradiation power and the irradiation distance was 22 ℃, was (30 ± 5)%, 60 W and 20 cm.

## 3. Results

**Figure 1:**
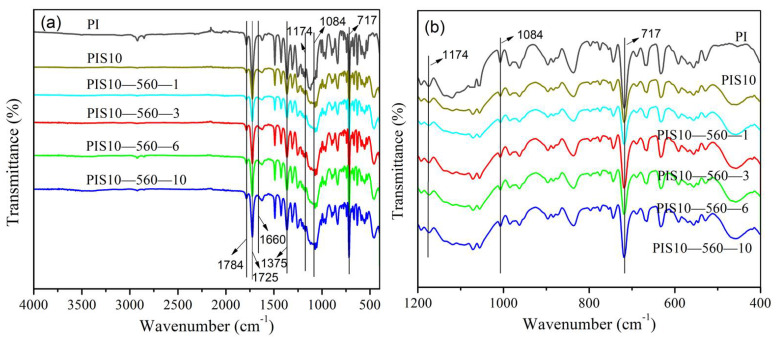
FTIR full spectra (**a**) and partical larging spectra (**b**) of PI, PIS10, PIS10-560-1, PIS10-560-3, PIS10-560-6 and PIS10-560-10.

The FTIR spectra of PI/SiO_2_ films prepared with various addition of KH-560 are shown in Figure 1. There is a C=O bending vibration absorption peak at 717 cm^−1^, an imine ring C-N-C stretching vibration absorption peak at 1375 cm^−1^, a C=O symmetrical extensional vibration absorption peak at 1725 cm^−1^ and a C=O asymmetrical extensional vibration absorption peak at 1784 cm^−1^ in the spectrum of each composite film. All samples did not exhibit the absorption peak of amide bond (1660 cm^−1^), suggesting the completely thermal imidization procedure [21,22].

As shown in Figure 1a, after adding SiO_2_, the FTIR spectra of the composite film show an obvious discrepancy between 1000 and 1100 cm^−1^. The FTIR spectrum of PI has no characteristic absorption peak, while the FTIR spectra of PI/SiO_2_ composite material have a characteristic absorption peak of the Si-O-Si bond (1084 cm^−1^), and an asymmetric stretch vibration absorption peak of the cyclic Si-O-Si bond (1174 cm^−1^). This demonstrates that the two phases in the system are connected to each other via chemical bonds, generating a cross-linked network construction, that is, a composite structure of PI/SiO_2_ strong interaction. Comparing the composite film materials with different dosages of silane coupling agent, it is found that the overall peak positions of the FTIR spectra are consistent, indicating that their chemical structures are consistent. The FTIR spectra are further enlarged locally, as shown in Figure 1b. The absorption peaks of the four composite films (PIS10-560-1, PIS10-560-3, PIS10-560-6, PIS10-560-10) with different addition amounts of silane coupling agent KH-560 are almost the same. Furthermore, the peak intensities at 1084 cm^−1^ and 1174 cm^−1^ are almost the same. The above two peaks correspond to the characteristic absorption peak and the asymmetric stretching vibration absorption peak of the Si-O-Si bond, suggesting that the amount of KH-560 does not affect the chemical bonding between two phases in the composite. The intensity of the corresponding peak at 717 cm^−1^ gradually improves with the increase of KH-560. This peak ascribes to the bending vibration of C=O. This could be ascribed to the generation of a hydrogen bond between KH-560 and PAA, which enhances the degree of polarization of the chemical bond [23,24].

**Figure 2:**
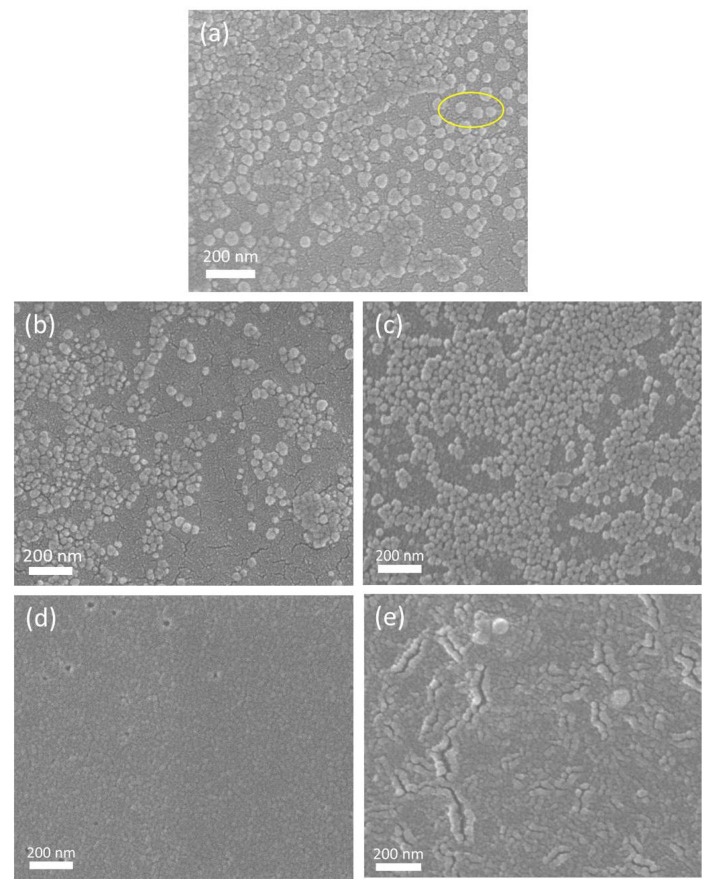
SEM images of PIS10 (**a**), PIS10-560-1 (**b**), PIS10-560-3 (**c**), PIS10-560-6 (**d**), PIS10-560-10 (**e**).

The SEM images of the PI/SiO_2_ composite films are shown in Figure 2. The small particles with white outlines in the picture are nano-SiO_2_ particles, and the dark gray background is a polyimide matrix. It can be seen that the organic–inorganic two-phase interface in the PIS10 composite film material without silane coupling agent is very clear, and the two-phase crosslinking is not enough. However, the interface between the two phases of the composite film material using the silane coupling agent KH-560 is relatively blurred. Because the addition of KH-560 offers abundant bonding points for the bonding between two phases, benefiting enhances the compatibility between two phases and makes the interface between the two phases relatively blurred. In addition, after adding KH-560, the particle size of the SiO_2_ nanoparticles is significantly reduced. Furthermore, a network structure gradually forms, in which SiO_2_ nanoparticles are cross-linked with polyimide and uniformly dispersed in it.

When a coupling agent KH-560 is used, the epoxy group at the end of the KH-560 molecule is hydrolyzed to form a hydroxyl group. Some hydroxyl groups undergo an esterification reaction with the carboxyl group of PAA. Some hydroxyl group forms a hydrogen bond with the carbonyl group of PAA. During thermal imidization, PAA undergoes a ring-closure reaction of dehydration and dealcoholization, decreasing the molecular weight and the viscosity of the system, which is more conducive to the dispersion of SiO_2_ in PI. In addition, the decrease of viscosity increases the freedom of PI molecular chain movement, which also increases the chance of reaction with the inorganic phase. The abundant Si-OH on the surface of SiO_2_ has a strong adsorption effect on the organic phase. As depicted in Figure 2b,c, the size of the SiO_2_ gradually reduces with the increase of KH-560. This is because the generated SiO_2_ and the PI matrix penetrate each other, and the surface of the SiO_2_ is coated by the organic phase, serving as a physical barrier and hindering SiO_2_ to agglomerate. Therefore, the addition of KH-560 enhances the compatibility of the two phases and inhibits the agglomeration of SiO_2_. Nevertheless, when the added amount of KH-560 is too high, as shown in Figure 2d,e, obvious agglomeration and cracks appear on the surface of the film, and the size of SiO_2_ and the distribution are not homogeneous.

**Figure 3:**
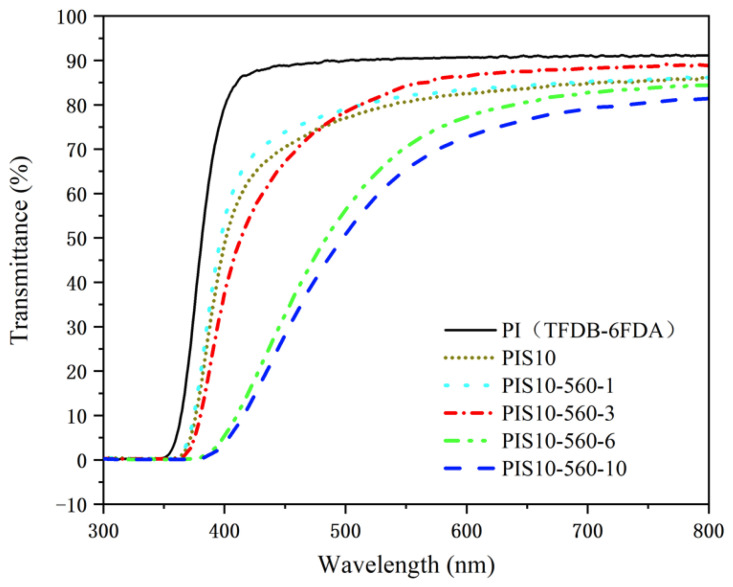
The transmittance of PI, PIS10, PIS10-560-1, PIS10-560-3, PIS10-560-6 and PIS10-560-10.

The optical transmittance spectra of PI/SiO_2_ films are displayed in Figure 3. The transmittance of the PI film (obtained from TFDB-6FDA) at about 400 nm is higher than 85%, and the average transmittance in the visible range can reach up to 91.4%. However, the traditional PI film (obtained from ODA-PDMA) has a transmittance of almost zero at about 400 nm, and the average transmittance in the optical range can only arrive at 65% [21,22,23]. After using the in situ polymerization method to incorporate 10 wt% SiO_2_ nanoparticles into the PI matrix (TFDB-6FDA system), the transmittance of all films in the 350–800 nm wavelength range significantly decreases. When the addition of KH-560 is 1%, the transmittance of PIS10-560-1 film is slightly higher than that of PIS10 in the wavelength range of about 370–800 nm. The transmittance spectrum of PIS10-560-3 intersects with PIS10 at 475 nm. At this point, the transmittance of the former on the left is lower than that of the latter, and on the right, it is higher than that of the latter. Furthermore, to increase the additive amount of coupling agent, the transmittance of PIS10-560-6 and PIS10-560-10 films decreased significantly. In addition, with the addition of nanoparticles and the increased amount of coupling agent, the cut-off wavelength of the transmittance of the composite film increased significantly. The transmittance of PIS10, PIS10-560-1, PIS10-560-3, PIS10-560-6 and PIS10-560-10 at around 500 nm are 77.5%, 78.5%, 78%, 51.5%, 49.6%, respectively. The average transmittance in the wavelength range of 380–780 nm is 55.7%, 58.2%, 63.6%, 61.3% and 56.2%, respectively. Obviously, as the amount of KH-560 added PIS10-560-3 intersects with PIS10 at 475 nm. At this point, the transmittance of the former on the left is lower than that of the latter, and on the right, it is higher than that of the latter. Further to increase additive amount of coupling agent, the transmittance of PIS10-560-6 and PIS10-560-10 films decreased significantly. In addition, with the addition of nanoparticles and the increased amount of coupling agent, the cut-off wavelength of the transmittance of the composite film increased significantly. The transmittance of PIS10, PIS10-560-1, PIS10-560-3, PIS10-560-6 and PIS10-560-10 at around 500 nm are 77.5%, 78.5%, 78%, 51.5%, 49.6%, respectively. The average transmittance in the wavelength range of 380–780 nm is 55.7%, 58.2%, 63.6%, 61.3% and 56.2%, respectively. As the additive amount of KH-560 increases, the transmittance of the composite film first increases and then decreases.

Under the same doping amount of SiO_2_ nanoparticles, the permeability performance of samples with lower coupling agent dosage (1%, 3%) is significantly improved than that of samples without coupling agent. Furthermore, The improvement of the sample PIS10-560-3 obtained by using 3% KH-560 is even more significant. When further improving the amount of KH-560, the transmittance of the samples decreased significantly. Because the added amount of KH-560 enhances the cross-linking between SiO_2_ and PI matrix. While the amount of KH-560 is low, the inorganic phase SiO_2_ can be tightly combined with the PI matrix, making the SiO_2_ particles difficult to agglomerate. The smaller particle size is beneficial to the increase of the transmittance of the film. While the amount of KH-560 is higher, the hydrogen bond is formed after the esterification reaction of the hydroxyl group in the silane coupling agent KH-560, which will form a thicker film on the surface of the SiO_2_ particles, as shown in Figure 2d,e. The binding capacity between the inorganic phase and the organic phase is greatly reduced due to its barrier. Furthermore, the transmittance of the PI/SiO_2_ reduces due to the film becomes thick [22].

**Figure 4:**
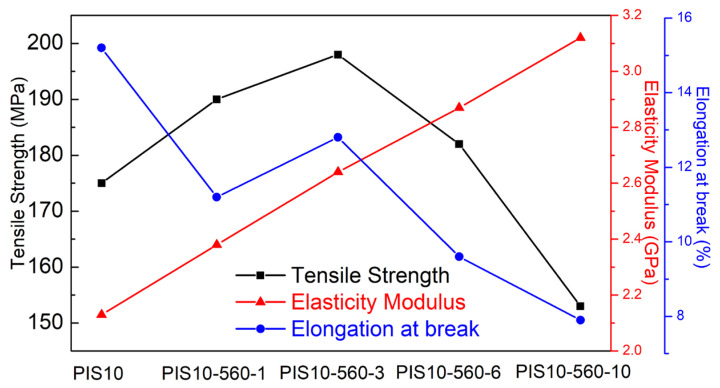
The mechanical properties of PIS10, PIS10-560-1, PIS10-560-3, PIS10-560-6 and PIS10-560-10.

For the mechanical performances of polymer composites, the three important indicators of the are elongation at break, tensile strength and elasticity modulus. The mechanical property data of the sample is displayed in Figure 4. Compared with PIS10,

When the SiO_2_ content is the same, the tensile strength first increases and then decreases with the enhanced addition of KH-560. The elastic modulus increases with the increased amount of KH-560. When the additive amount of KH-560 is low, the formed SiO_2_ particles are smaller and are evenly distributed in the PI matrix. The specific surface area is large and the adsorption for PI molecular chains is strong, thereby improving the elastic modulus and tensile strength of the film, especially when the added amount of silane coupling agent KH-560 is 3%. While the additive amount of KH-560 is too high, obvious agglomeration and cracks appear, as shown in Figure 2d,e. The size and distribution of the SiO_2_ are not uniform, leading to the diminution of the valid contact of the two phases and the receding of the interaction, decreasing its mechanical properties. In addition, the elongation at the breaking of the film gradually decreases with the increased amount of KH-560. This could be because a large amount of KH-560 destroys the molecular chain structure of polyimide, decreasing the force between imine molecules. When the additive amount of KH-560 firstly increases from 1% to 3% and then increases to 6% and 10%, the elongation at break first increased and then decreased. These phenomena are related to the interface interaction between polyimide and silica. After adding the silane coupling agent KH-560, the cross-linking of polyimide and silica is beneficial to the increase of tensile strength. However, as the dosage of silane coupling increases, the hardness of the polyimide composite film increases and the flexibility decreases. The composite films obtained with a moderate amount of KH-560 (3%) has better dispersibility and interfacial adhesion compared with other composite materials obtained by KH-560. Under the action of tensile stress, the SiO_2_ nanoparticles become the receptors of tensile force. Once the tensile stress of the composite material exceeds the marginal value, the breakdown of the composite material is caused by internal failure. If the amount of KH-560 is too high or too low, the toughness of the composite material will decrease, and many defects will be generated in the mesophase, thus making the damage of the composite material easier. The elongation at break is one of the important indexes to measure the relative strength of the film. A film with a large elongation at break has a softer hand and can cushion the force. Based on the above description, with the addition of KH-560, the hardness of the polyimide film increases and the flexibility decreases, so the PIS10 shows the largest elongation at break.

**Figure 5:**
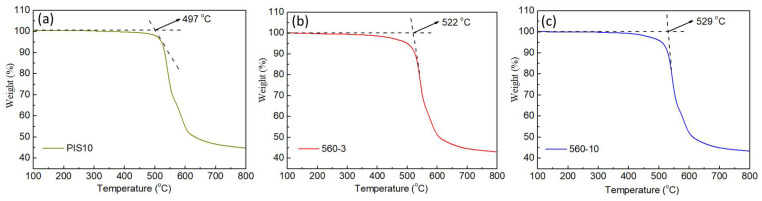
TGA curves of PIS10 (**a**), PIS10-560-3 (**b**) and PIS10-560-10 (**c**).

The TGA curves of three PI/SiO_2_ composite films are displayed in Figure 5. Among them, the thermal destruction temperature of the PIS10 is 497 °C (Figure 5a). The thermal destruction temperature of PIS10-560-3 and PIS10-560-10 are 522 °C and 529 °C, respectively, as shown in Figure 5b,c, respectively, which is more thermal stability than that of pure PI in the literature [25,26]. In addition, at 700 °C, the residual rate of the PIS10 is 46.7%, and the residual rates of samples PIS10-560-3 and PIS10-560-10 are 44.6% and 45%, respectively.

It can be seen that the heat stability of the film enhances slightly with the improved with the addition of KH-560. This is because the epoxy group at the end of the KH-560 molecule is hydrolyzed to form a hydroxyl group, and a part of the hydroxyl group undergoes an esterification reaction with the carboxyl group in the PAA molecule, decreasing the molecular weight and the viscosity of the system, which is more conducive to the dispersion of the inorganic phase in the PI. The viscosity decreases and the degree of freedom of PI molecular chain movement increases, which also increases the chance of reaction with the inorganic phase. Abundant Si-OH on the surface of the SiO_2_ nanoparticles has a strong adsorption effect on the organic phase, which inhibits the thermal vibration of polyimide to a certain extent. Thereby it is beneficial to enhance heat stability. The coupling agent KH-560 connects the two phases through van der Waals force and hydrogen bonding, which also improves the thermal stability to a certain extent.

It is well known that the energy of ultraviolet light is adequate to break up the chemical bonds and cause the cover of polymer materials to crack or combined reaction, which decreases the mechanical properties of the material [23,24,25,26]. As shown in Table 1, when KH-560 is affiliated, after UV irradiation the mechanical properties of the film change a lot. The attenuation rate of the tensile strength of PIS10-560-1, PIS10-560-3, PIS10-560-6, and PIS10-560-10 decreased from 13.71% of PIS10 film to 13.16%, 10.65% and then increased to 13.73%, 18.95%. The attenuation rate of elastic modulus decreased from 7.04% of PIS10 film to 6.72%, 6.44% and then increased to 7.32%, 9.29%. Furthermore, the attenuation rate of elongation at break increased slightly from 9.21% of PIS10 film to 10.09 %, then dropped to 8.59%, and then further increased to 11.46% and 16.46%.

The aforementioned results fully illustrate that the introduction of KH-560 has availably resisted the influence of ultraviolet radiation on the PI/SiO_2_ film when the added amount is low. When the added amount is 3%, the ability to resist ultraviolet radiation is the strongest. At higher doses, its ability to resist ultraviolet radiation is weaker. After irradiation, the free radicals are generated. In the combining procedure of between free radicals and protons, cross-linking or chain scission could happen. Once free radicals combine with protons, the molecular link cracks from a long chain to a correspondingly short segment, and a decomposition reaction happens.

While two free radicals are combined, the two links change into a longer chain fragment, and a cross-linking reaction happens. Breaking will reduce the elongation at break, elastic modulus and tensile strength. While cross-linking will improve the elastic modulus and tensile strength, and decrease the elongation at break. When the tensile strength, elongation at break, and elastic modulus reduce contemporarily, it suggests that cross-linking and intramolecular fracture occur at the same time, and fracture decomposition plays a major role. Otherwise, cross-linking plays a major role. Without adding any silane coupling agent, SiO_2_ can not be connected to the PI in the shape of chemical bonds. The space occupied by the silanol aggregates that are severely agglomerated during the phase inversion process becomes a defect in the membrane structure, so the mechanical strength of the PIS10 is poor. The silane coupling agent KH-560 connects the organic/inorganic two phases through van der Waals forces and hydrogen bonds. When the dosage is low, the addition of KH-560 increases the dispersion of SiO_2_ in the PI matrix due to the connection influence of KH-560. The defects of the film material are reduced, and the elastic modulus and tensile strength are improved. When the added amount is higher, the silane coupling agent is not resistant to radiation, so the above-mentioned connection effect is weakened, thereby reducing the mechanical properties.

As shown in Table S2, after UV irradiation, the transmittance of PIS10 film and PI/SiO_2_ composite film obtained from different amounts of silane coupling agent has a slight attenuation. Nevertheless, the discrepancy in attenuation rate is very small. The attenuation rate of the composite film obtained from different silane coupling agent is slightly lower, suggesting that the addition of KH-560 has little effect on the permeability of PIS10 after irradiation.

## 4. Conclusions

In this work, TFDB and 6FDA were used as monomers to prepare PI/SiO_2_ films by in situ polymerization. The effect of the addition of KH-560 on the structure and properties of the film was studied. The results showed that the thermal imidization reaction of the PI/SiO_2_ film was entire, and the inorganic-organic phase generated a cross-linked reticular formation. FTIR, SEM, TGA, simulated ultraviolet radiation and ultraviolet-visible photometer were used for characterizing the structure, optical, mechanical performances and resistance to UV radiation performance of the composite film. When the coupling agent is not added, the addition of SiO_2_ reduces the transmittance of the PI sample. Comparing the components with the same SiO_2_ doping amount, the transmittance of the sample with KH-560 in the visible light range is higher than that of without KH-560. At the same SiO_2_ content, the tensile strength of the film first enhanced and then reduced with the addition of KH-560, while the elastic modulus continued to increase, and the elongation at break gradually reduced. The thermal stability of the composite film firstly improves and then reduces. The mechanical performances of the composite film against ultraviolet radiation also firstly increase and then decrease. With the improvement of the coupling agent, the attenuation of elastic modulus, the elongation at break and tensile strength of the film firstly decreases and then increases. The optimizing addition amount of KH-560 is 3%. The addition of KH-560 also inhibited the attenuation of the transmittance of the composite film after irradiation.

## Figures and Tables

**Table 1:** The mechanical properties of PI/SiO_2_ films before and after irradiation.

Sample	Tensile Strength (Mpa)			Elasticity Modulus (Gpa)			Elongation at Break (%)		
	Before	After	Attenuation Rate (%)	Before	After	Attenuation Rate (%)	Before	After	Attenuation Rate (%)
PIS10	175	151	13.71	2.13	1.98	7.04	15.2	13.8	9.21
560-1	190	165	13.16	2.38	2.22	6.72	11.2	10.07	10.09
560-3	198	176.9	10.65	2.64	2.47	6.44	12.8	11.7	8.59
560-6	182	157	13.73	2.87	2.66	7.32	9.6	8.5	11.46
560-10	153	124	18.95	3.12	2.83	9.29	7.9	6.6	16.46

## Data Availability

The data presented in this study are available on request from the corresponding author.

## Supplementary Materials

The following are available online at https://www.mdpi.com/article/10.3390/nano12020286/s1, Figure S1: The molecular structure of KH-560. Figure S2: The schematical diagram of PI/SiO_2_ films. Table S1: Composition of PI/SiO_2_ composite film obtained by using different additive amount of silane coupling agent. Table S2: The transmittance of PI/SiO_2_ films before and after irradiation.
