# Supplementary material for: The Effect of Silane Coupling Agent on the Texture and Properties of In Situ Synthesized PI/SiO2 Nanocomposite Film
Source: Nanomaterials (Basel). 2022 Jan 17;12(2):286. doi: 10.3390/nano12020286 (PMC8778991; doi:10.3390/nano12020286)
Supplement: Supplementary file 1 [file nanomaterials-12-00286-s001.zip › nanomaterials-1528466-supplementary.pdf]

nanomaterials

MDPI

Supplementary Materials

The Effect of Silane Coupling Agent on the Texture and Properties of In Situ Synthesized PI/SiO2 Nanocomposite Film

Jindong Huang [1,2], Hong Chen [1], Guanglu Zhang [1,2], Xiaowei Fan [1,3] and Juncheng Liu [1,*]

[1] School of Materials Science and Engineering, Tiangong University, Tianjin 300387, China; jdhuang@tiangong.edu.cn (J.H.); chen393994@gmail.com (H.C.); zgltg@tiangong.edu.cn (G.Z.); xiaowei_fan@126.com (X.F.)
[2] School of Physical Science and Technology, Tiangong University, Tianjin 300387, China
[3] Tianjin SYP Engineering Glass Co., Ltd., Tianjin 300402, China
[*] Correspondence: jchliu@tiangong.edu.cn; Tel.: +86-(0)-22-83-955-811

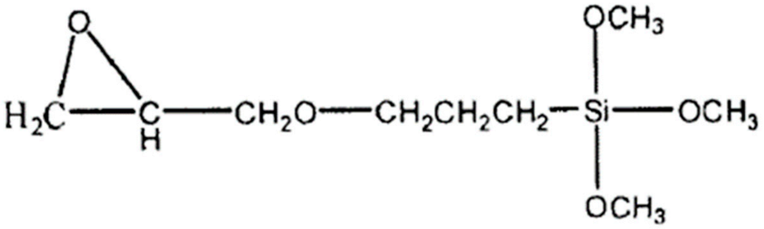

Figure S1. The molecular structure of KH-560.

Table S1. Composition of PI/SiO_2 composite film obtained by using different additive amount of silane coupling agent.

Sample	Mass (g)				Coupling agent accounts for SiO_2 mass percentage (%)	SiO2 mass percentage (%)	Solid content of PAA solution (%)
	**SiO2**	**TFDB**	6FDA	DMAc			
**PI**	**0**	**3.203**	4.532	77.735	0	0	9.95
PIS10	0.877	3.210	4.531	77.743	0	10.17	9.98
PIS10-560-1	0.876	3.206	4.530	77.741	1.02	10.17	9.95
PIS10-560-3	0.878	3.207	4.529	77.740	3.05	10.19	9.95
PIS10-560-6	0.878	3.209	4.532	77.743	6.01	10.18	9.96
PIS10-560-10	0.878	3.208	4.531	77.742	10.03	10.19	9.95

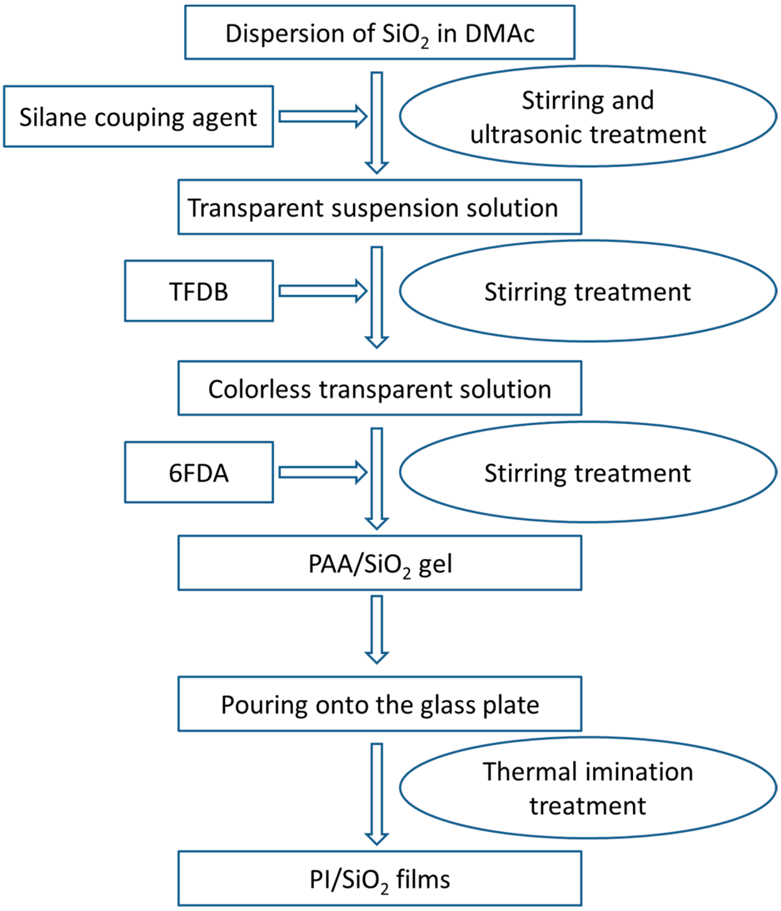

Figure S2. The schematical diagram of PI/SiO_2 films.

Table S2. The transmittance of PI/SiO_2 films before and after irradiation.

**Sample**	**Maximum Transmittance (%)**		**Attenuation Rate (%)**
	Before	After	
**PIS10**	87.6	86.3	1.48
PIS10-560-1	86.6	85.4	1.38
PIS10-560-3	86.9	85.7	1.38
PIS10-560-5	85.2	84.1	1.29
PIS10-560-10	84.9	83.8	1.29
